# Early chest CT abnormalities to predict the subsequent occurrence of chronic lung allograft dysfunction

**DOI:** 10.1186/s13244-023-01509-3

**Published:** 2023-09-23

**Authors:** Paul Habert, Elsa Chetrit, Benjamin Coiffard, Fabienne Bregeon, Pascal Thomas, Anderson Loundou, Julien Bermudez, Martine Reynaud-Gaubert, Jean-Yves Gaubert

**Affiliations:** 1 Service de radiologie, Hôpital Nord, Chemin des Bourrely, 13015 Marseille, France; 2 Aix Marseille Univ, LIIE, Marseille, France; 3 Aix Marseille Univ, CERIMED, Marseille, France; 4 Centre de Ressources et de Compétences de la Mucoviscidose (CRCM) Adulte, AP-HM Hôpital Nord, 13015 Marseille, France; 5 APHM, Hôpital Nord, Explorations Fonctionnelles Respiratoires, Marseille, France; 6 Aix Marseille Univ, APHM, Microbes Evolution Phylogeny and Infections (MEPHI), IHU-Méditerranée Infection, Marseille, France; 7 Service de chirurgie thoracique, Hôpital Nord, chemin des Bourrely, 13015 Marseille, France; 8 Aix-Marseille Univ, - CEReSS UR3279-Health Service Research and Quality of Life Center, Marseille, France; 9 Department of Public Health, Assistance Publique - Hôpitaux de Marseille, Marseille, France; 10 Service de radiologie, La Timone Hôpital, 264 rue Saint Pierre, 13005 Marseille, France

**Keywords:** Lung transplantation, Follow-up studies, Graft rejection, X-Ray computed tomography

## Abstract

**Introduction:**

Chronic lung allograft dysfunction (CLAD) can take two forms: bronchiolitis obliterans syndrome (BOS) or restrictive allograft syndrome (RAS). The aim was to determine if chest-CT abnormalities after lung transplantation (LTx) could predict CLAD before respiratory functional deterioration.

**Materials and methods:**

This monocentric retrospective study analyzed consecutive patients who underwent LTx from January 2015 to December 2018. Initial CT post-LTx (CTi) and a follow-up CT at least 9 months post-LTx (CTf) were reviewed. CLAD was defined as a persistent respiratory functional decline (> 20% of basal FEV_1_) outside acute episode. A Cox regression was performed in univariate, then in multivariate analysis (including features with *p* < 0.01 in univariate or of clinical importance) to determine risk factors for CLAD. Subgroup analyses were made for BOS, RAS, and death.

**Results:**

Among 118 LTx patients (median (min–max) 47 (18–68) years), 25 developed CLAD during follow-up (19 BOS). The median time to CLAD since LTx was 570 days [150–1770]. Moderate pulmonary artery stenosis (30–50%) was associated with the occurrence of CLAD on CTi (hazard ratio HR = 4.6, CI [1.6–13.2]) and consolidations and pleural effusion on CTf (HR = 2.6, CI [1.3–4.9] and HR = 4.5, CI [1.5–13.6] respectively). The presence of mosaic attenuation (HR = 4.1, CI [1.4–12.5]), consolidations (HR = 2.6, CI [1.3–5.4]), and pleural effusions (*p* = 0.01, HR = 5.7, CI [1.4–22.3]) were risk factors for BOS on CTf. The consolidations (*p* = 0.029) and pleural effusions (*p* = 0.001) were risk factors for death on CTf.

**Conclusions:**

CTi and CTf in the monitoring of LTx patients could predict CLAD. Moderate pulmonary artery stenosis, mosaic pattern, parenchyma condensations, and pleural effusions were risk factors for CLAD.

**Critical relevance statement:**

There is a potential predictive role of chest CT in the follow-up of LTx patients for chronic lung allograft dysfunction (CLAD). Early chest CT should focus on pulmonary artery stenosis (risk factor for CLAD in this study). During the follow-up (at least 9 months post-LTx), parenchymal consolidations and pleural effusions were shown to be risk factors for CLAD, and death in subgroup analyses.

**Key points:**

• Pulmonary artery stenosis (30–50%) on initial chest-CT following lung transplantation predicts CLAD HR = 4.5; CI [1.6–13.2].

• Pleural effusion and consolidations 1 year after lung transplantation predict CLAD and death.

• Early evaluation of lung transplanted patients should evaluate pulmonary artery anastomosis.

**Graphical Abstract:**

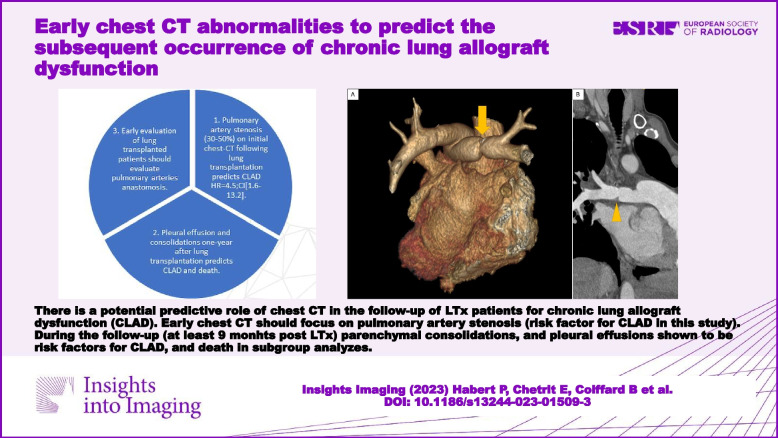

**Supplementary Information:**

The online version contains supplementary material available at 10.1186/s13244-023-01509-3.

## Introduction

Lung transplantation (LTx) is a recognized treatment option for selected patients with end-stage chronic respiratory disease. Since the beginning, there has been a significant and steady increase in lung transplant activity, and post-transplant survival outcomes have improved [1]. However, medium- and long-term survival remains limited by the occurrence of chronic lung allograft dysfunction (CLAD). First described only as bronchiolitis obliterans syndrome (BOS), an obstructive syndrome, chronic rejection now comprises two phenotypes with different functional and evolutionary profiles. BOS is now distinguished from restrictive allograft syndrome (RAS) with a restrictive functional profile [2, 3]. A third mixed phenotype was recently described, defined as a restrictive physiology with persistent CT opacities after initial BOS [4]. According to the latest published report, the prevalence of BOS is estimated to reach nearly 47% of 5-year survivors and is the leading cause of mortality at 3 years post-transplantation [1].

Histologically, BOS involves obliteration of the distal small airways by an inflammatory and fibroproliferative process [5]. RAS is characterized histologically by various stages of diffuse alveolar damage and extensive fibrosis in the peri-alveolar space, visceral pleura, and inter-lobular septa, with or without scattered BOS lesions. Due to the low sensitivity of trans-bronchial biopsies [6, 7] and the invasive nature of surgical lung biopsies, the diagnosis of CLAD is still based on functional criteria. Apart from patients sensitive to azithromycin [2, 8], no significant reversibility could be obtained. Finally, it has been shown that early diagnosis and management of BOS may improve long-term survival after LTx [9, 10].

The results of several studies suggest that CT has an important role in detecting BOS in lung-transplanted patients [11–14]. The potential role of CT scanning before the onset of respiratory deterioration has been evaluated in several studies [3, 15–18] but has not yet been proven. This is the most important period, as early identification of patients at risk of progressing to CLAD would allow earlier adaptation of management. The CT signs in proven BOS are those of bronchiolar obstruction: parenchymal distension with a mosaic appearance of the lung parenchyma, unmasked by air trapping on expiration series, in the pathologic locations. The airways are dilated and malacic, sometimes with thickening of the bronchial wall. Later, septal thickening and subpleural reticulation appear as signs of pulmonary fibrosis [19]. RAS has a poorer prognosis [20], limited to 6–18 months compared to 3–5 years in BOS [2, 21]. It is characterized on CT scan by lobar shrinking, predominantly in the upper lobes and more precisely in the apices, with interstitial reticulations, condensation, ground glass, and traction bronchiectasis (usually absent in patients with BOS) [2, 20].

The main objective of this study was to determine if early CT abnormalities could predict CLAD. The secondary objective was to determine if these abnormalities could predict death.

## Materials and methods

### Patients

This single-center, retrospective study collected consecutive adult patients who underwent mono or bi-pulmonary transplantation from January 2015 to December 2018. For all patients, initial chest CT (CTi) available 1 to 4 months after LTx, and follow-up CT (CTf) available 9 months to 2 years after LTx, were retrieved from the Picture Archiving Computing System of the institution. Patients who died in the first year after the LTx and those for whom imaging data were incomplete were excluded from the study. Institutional review board approval was obtained (Comité d’Ethique pour la Recherche en Imagerie Médicale n°CRM-2206-269).

### Immunosuppressive and anti-infectious prophylaxis regimens

In the study period, all recipients received a standardized immunosuppressive regimen in accordance with our institutional protocol. Induction therapy consisted of intravenous administration of anti-thymocyte globulin (1.5 mg/kg/day) for 3 days, associated with high-dose methylprednisolone (7.5 mg/kg before each lung implantation). Standard triple maintenance immunosuppressive regimen consisted of intravenous cyclosporine administered immediately after LTx (to obtain a steady-state serum concentration between 300 and 400 ng/ml) and then switched by oral tacrolimus as soon as possible (to maintain trough blood levels between 12 and 15 ng/ml during the first 3 months and around 10–12 ng/ml thereafter), mycophenolate mofetil, and steroids (1 mg/kg/day of prednisone) tapered to 0.5 mg/kg/day over the first month and then progressively tapered over the first year to 5 mg per day. Postoperatively, transplant recipients received a prophylactic antibiotic treatment according to their preoperative and/or concomitant infectious status for at least 7 days. Seropositive CMV recipients and higher-risk CMV-mismatched recipients (donor positive and recipient negative) received prophylactic IV ganciclovir or oral valganciclovir as soon as possible, for the entire study period.

### Respiratory function tests

Respiratory parameters of lung transplant patients were assessed from functional respiratory tests on two different measurements separated by at least 3 weeks. CLAD is defined as a decrease in forced expiratory volume in 1 s (FEV_1_) < 80%, from the average of the two best values obtained post-LTx, apart from any acute event [22]. BOS is defined as a persistent decline in FEV_1_ > 20% on two consecutive measurements and associated with a non-reversible obstructive ventilatory disorder (FEV_1_/FVC < 70%). RAS is defined by (1) a persistent ≥ 20% decline in FEV1 (+/−FVC) compared with the reference or baseline value; (2) a decrease in total lung capacity (TLC) to ≤ 90% compared with baseline, defined as the average of the 2 measurements obtained at the same time as or very near to the best 2 post-operative FEV_1_ measurements; and (3) the presence of persistent, opacities on chest imaging [22, 23]. When lung volumes cannot be measured specifically, restrictive lung injury can be estimated from a decrease in vital capacity (FVC) with a normal or increased FEV_1_/FVC ratio. The measured TLC of the recipient in pre-LTx was reported as well as the best FEV_1_ value obtained in post-LTx.

### Monitoring in imaging

All scans were performed on two machines (Somatom Definition 64, Siemens Healthcare, Erlangen, Germany, or lightspeed VCT, GE Healthcare, Milwaukee, WI, USA). All CTi were contrast-enhanced CT to analyze pulmonary arteries, with low degree of inspiration to avoid the Valsalva effect, from the bases to the apices, with a nominal slice thickness of 0.6 or 0.75 mm and reconstructions spaced at 0.7- to 1-mm intervals and a voltage of 100 kV. For CTf, inspiratory images were acquired in deep forced inspiration, from the bases to the apices, with a nominal slice thickness of 0.6 or 0.75 mm and reconstructions spaced at 0.7- to 1-mm intervals. Images were acquired with a voltage of 120 kV. All CT has automatic modulation for tube current (mA) and was reconstructed in mediastinal and parenchymal windows. Expiratory CT was performed at the end of the expiration with the same parameters and a 20-kV reduction of the tube voltage.

### Thoracic CT evaluation

CT results were evaluated in 2021, by two chest radiologists (P.H. and E.C.) with 8 and 5 years of experience in thoracic imaging, respectively. In case of disagreement, a third radiologist (JY.G.) with 30 years of experience resolved the issue. The readers had no information about patient outcomes (CLAD, death). Abnormalities were assessed according to the Fleischner Society [24], using semi-quantitative scores and based on descriptions from previous studies [3, 11, 15]. For each patient, the CTi was analyzed first, followed by the CTf. The criteria assessed are detailed in Table 1. Lung volumes were measured using workstation-based quantification software (Thoracic VCare, Workstation ADW, GE; Milwaukee).

**Table 1:** Scores assessing the extent and severity of CT abnormalities

		0	1	2	3
Liquid pleural effusion		absent	mild	moderate	high
Pneumothorax		absent	mild	moderate	high
Bronchiectasis (per lobe)		absent	cylinder	varicose	cystic
Bronchial anastomotic stenosis		< 50%	50% to 65%	> 65%	
Bronchial anastomotic dehiscence		absent	moderate < 1 cm	severe > 1 cm	
Bronchial wall thickening		absent	mild	severe	
Anastomotic granuloma		yes	no		
Mosaic attenuation		yes	no		
Pulmonary embolism		absent	>4^th^ order	segmental	lobar
Arterial anastomotic stenosis		absent	< 50%	> 50%	
	0	1	2	3	4
GGO	absent	< 10 lobules	1 to 3 segments	4 to 6 segments	diffuse
Consolidations	absent	< 10 lobules	1 to 3 segments	4 to 6 segments	diffuse
retractile consolidations	absent	< 10 lobules	1 to 3 segments	4 to 6 segments	diffuse
Interstitial opacities	absent	< 10 lobules	1 to 3 segments	4 to 6 segments	diffuse
Air trapping (per lobe)	< 25%	25% to 50%	50% to 75%	> 75%	
Tree in bud nodules	0 to 20 segments involved				
GGO alveolar nodules					
Proximal bronchiectasis					
Distal bronchiectasis					
Bronchial wall thickening					
Mucous plugs					

*GGO* Ground-glass opacities

Proximal bronchiectasis was considered significant when the internal diameter of the bronchus was greater than 1.5 times the diameter of the accompanying artery. Bronchiectasis was classified as cylindrical, varicose, or cystic, with increasing severity assessed for each lobe [24]. Distal bronchiectasis was defined by the visibility of bronchioles within 2 cm of the pleura. The severity of bronchial wall thickening was assessed visually and classified as absent/moderate/severe: the most severe score was recorded for both lungs. The extent of tree-in-bud micronodules, ground-glass opacities, alveolar nodules, proximal and distal bronchiectasis, bronchial wall thickening (regardless of the severity of involvement), and mucoid plugs was assessed by the number of affected segments per transplanted lung (with a maximum score of 10 per transplanted lung for each CT abnormality). Mosaic attenuation was classified as present or absent, using minimum-intensity projection mode, on inspiratory CT. The presence of mosaic attenuation restricted to lower areas was not considered significant. Ground-glass opacities, retractile consolidations (area of consolidation with lung volume loss, equivalent to atelectasis), non-retractile consolidations (area of consolidation without lung volume loss), and interstitial opacities (non-septal lines) were assessed semi-quantitatively according to their extent, with a score ranging from 0 to 4. Bronchial anastomotic stenosis was considered significant above 50% [25]. The presence of a caliber mismatch between the donor and recipient arteries, or a true stenosis centered on the anastomosis, was assessed by measuring the ratio of the vessel diameter to the peak stenosis diameter and classified as absent or mild irregularity, moderate stenosis between 30% and 50%, and severe stenosis >50%. If a pulmonary embolism was present, the severity was recorded according to the distality of the involvement. We assessed the presence or absence of bronchomalacia (reduction of more than 50% of the stem bronchus or trachea lumen diameter on expiratory CT) and the extent of air trapping on the expiratory CT (air trapping over less than 25% of the parenchyma was considered normal) [18]. Minimum intensity projection mode was used to identify trapping. Each lobe was analyzed separately with an air trapping score per lobe ranging from 0 to 3. The scores for each lobe (six lobes considering the lingula as the 6^th^ lobe) were summed for a maximum total score of 18. For statistical analyses in mono-pulmonary transplantation, only the transplanted lung was assessed, and the score was doubled [11].

### Statistical analysis

Statistical analyses were performed using IBM SPSS Statistics version 20.0 (Inc., IL., USA). Continuous variables are presented as median (minimum–maximum). Categorical variables are presented as numbers and percentages. The relationship between categorical variables was assessed using Pearson’s chi-square test or Fisher’s exact test if the theoretical numbers were less than 5. The relationship between a qualitative variable and a quantitative variable was assessed using the Student *t*-test or the non-parametric Mann-Whitney test. The impact of certain parameters on events that occur over time was assessed using the Cox model. Survival curves for these events were constructed using the Kaplan-Meier method, and the curves were compared using the log-rank test. Variables with a *p*-value < 0.10 in the univariate analysis and those of clinical relevance were introduced in the multivariate analysis based on the Cox model. The relative risks are presented with their 95% confidence intervals (CI). For all tests, the threshold for statistical significance was set at *p* < 0.05.

## Results

### Population

**Fig. 1:**
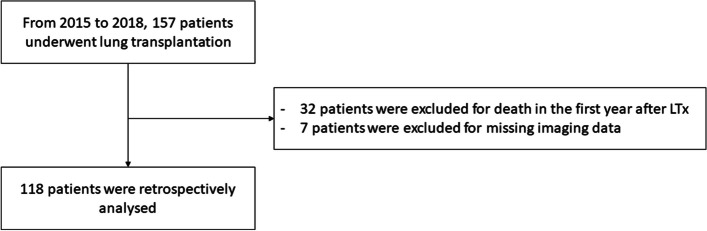
Flow chart of the study

This study reviewed 157 patients with mono or bi-pulmonary transplantation. Thirty-two patients were excluded due to death in the first year of follow-up. Seven other patients were excluded because of missing imaging data. A total of 118 patients had been retrospectively analyzed (Fig. 1), comprising 63 men (53.4%), with a median age of 47 years [18–68]. The median follow-up was 3.9 years [1.2–6.5]. Ninety-one patients (77.1%) received a bi-pulmonary transplantation; 27 patients (22.9%), a mono-pulmonary transplantation. The median donor PaO2/FiO2 was 417 [217–604] and graft ischemic time 327 min [16–694]. Of the 118 patients included, 19 (15.7%) died during follow-up. The characteristics of the population are summarized in Table 2.

**Table 2:** Clinical characteristics of the study population

	*N* = 118
Age at LTx (years)	47 [18–68]
Recipient gender (male)	63 (53.4%)
Recipient size (m)	1.66 [1.46–1.87]
Theoretical recipient TLC (L)	5.79 [3.85–7.86]
Bi-pulmonary transplantation	91 (77.1%)
Mono-pulmonary transplantation	27 (22.9%)
Graft ischemic time (min)	327 [16–694]
Donor PaO_2_/FiO_2_	417 [217/604]
Donor gender (male)	65 (55.1%)
Donor size (m)	1.70 [1.52–1.92]
Theoretical donor TLC (L)	6.4 [3.1–8.3]
Recipient TLC before LTx (%)	103.4 [38.7–218.3]
FEV_1_ baseline (L)	2.5 [1.0, 4.9]
Ratio theoretical TLC donor/recipient	1.06 [0.62–1.90]
Etiology for LTx	
Emphysema	35 (29.7%)
Fibrosis	28 (23.7%)
Cystic fibrosis and bronchial dilatations	42 (35.6%)
New LTx for CLAD	6 (5.1%)
Others	7 (5.9%)
Reject type	
Stable	93 (78.8%)
CLAD	25 (21.2%)
BOS	19 (16.1%)
RAS	6 (5.1%)
Death	19 (15.7%)
Delay for chest CT after LTx (days)	
Initial (CTi)	47 [31–134]
Follow-up (CTf)	381 [298–742]
Follow-up duration (years)	3.9 [1.1–6.5]

Results given as median [min-max] or *n* (%). *BOS* Bronchial obstructive syndrome, *CLAD* Chronic lung allograft disease, *CT* Computed tomography, *LTx* Lung transplantation, *RAS* Restrictive allograft syndrome, *TLC* Total lung capacity

**Fig. 2:**
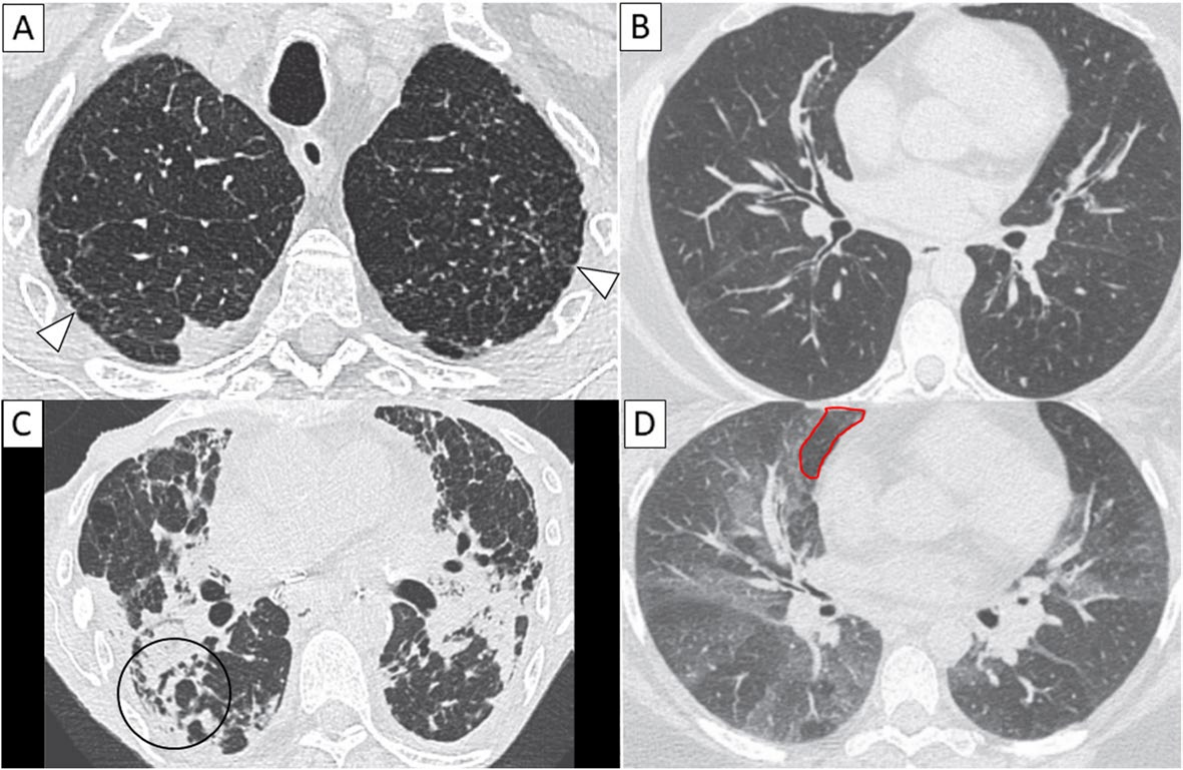
Unenhanced axial chest CT showing on the left side of the panel examples of RAS pattern (**A**, **C**), with subpleural reticular opacities (white arrowheads), volume loss, distortion, and traction bronchiectasis (black circle). On the right side of the panel, the images show examples of BOS pattern with a normal CT on deep inspiratory CT (**B**) and air trapping on the expiratory CT; an area is highlighted by a red circle (**D**)

Twenty-five patients (21%) developed CLAD: 6 RAS (5.1%) and 19 BOS (16.1%) (Fig. 2). One patient developed a BOS and then evolved to a RAS. The median time to CLAD since LTx was 570 days [90–1822]. The median time to BOS and RAS after LTx was 544 [179–1822] and 591 [90–934] days, respectively. The median time to CTi and CTf was 47 days [31–134] and 371 days [298–703], respectively. The median time from CTi and CTf to the functional diagnosis of BOS was 737 days [387–1805] and 421 days [65–1448].

### Multivariate analysis of the relation between CT data and the occurrence of CLAD

**Fig. 3:**
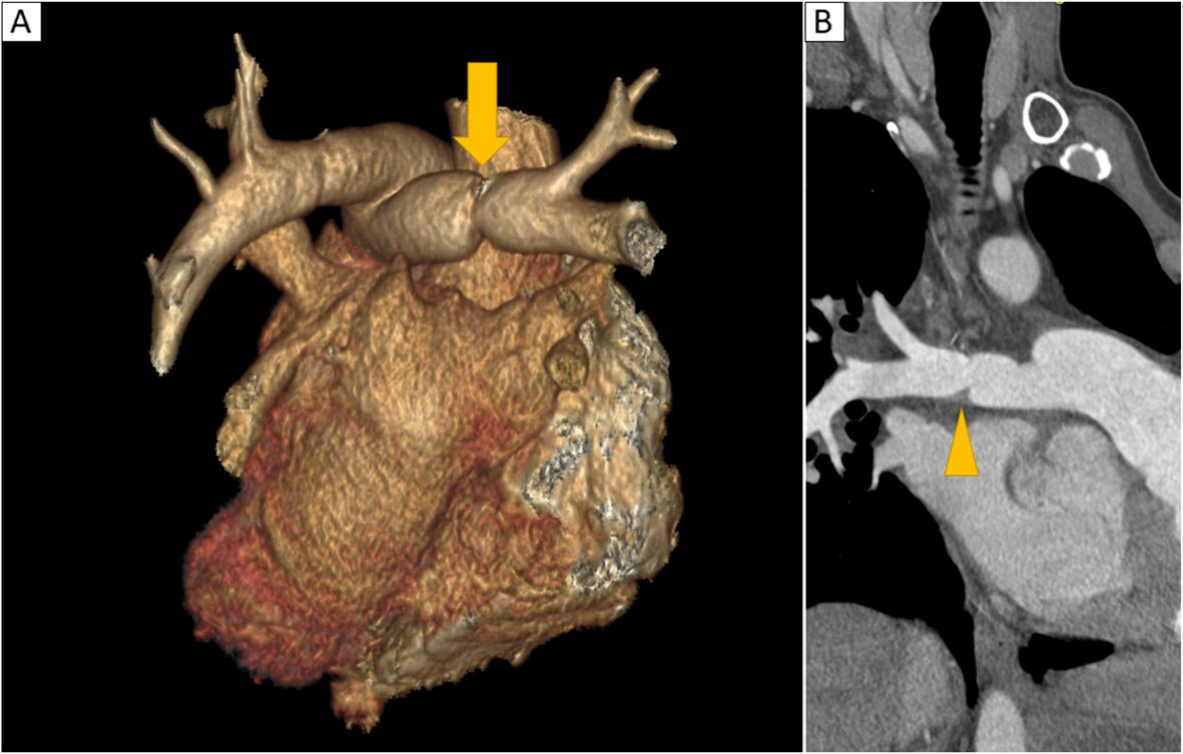
**A** Volume rendering of a posterior view of the heart showing moderate anastomotic stenosis of the right pulmonary artery (35%) (Orange arrow). **B** Same patient with contrast-enhanced chest CT, curvilinear reconstruction along the pulmonary artery from the right ventricle to the segmental arteries, showing the same stenosis (orange arrowhead)

Age, gender, etiology for LTx, consolidations, pleural effusion, and pulmonary artery stenosis were included in the multivariate analysis. On CTi, 11 cases of moderate anastomotic arterial stenosis (30 to 50%) occurred, of which 6 patients progressed to CLAD (54.5%), and this was a risk factor for CLAD (HR = 4.5; CI [1.6–13.2], *p* = 0.01) (Fig. 3). On CTf, pulmonary consolidation and pleural effusions were significantly associated with the occurrence of CLAD (HR = 2.6 [CI: 1.3–4.9], *p* = 0.01 and HR = 4.5 [CI: 1.5–13.6], *p* = 0.01, respectively) (Table 3).

**Table 3:** Multivariate analysis for the occurrence of CLAD and death

	**Parameters**	***p*-value**	**HR**	**CI 95%**
**Multivariate analysis for CLAD**				
Clinical	Age at LTx	0.76	1.0	0.9–1.1
	Gender	0.14	2.2	0.8–6.1
	Etiology for LTx	0.40		
CTi	Moderate arterial anastomosis stenosis	**0.01**	4.5	1.6–13.2
CTf	Consolidations (> 4 segments)	**0.002**	2.9	1.2–7.4
	Pleural effusion	**0.01**	2.8	1.5–5.4
**Multivariate analysis for death**				
Clinical	Age at LTx	0.96	1.00	0.9–1.1
CTf	Pleural effusions	**< 0.001**	9.8	2.7–35.7
	Consolidations (1–3 segments)	**0.01**	8.2	1.5–43.3

*CLAD* Chronic lung allograft disease, *CT* Computed tomography, *LTx* Lung transplantation, *RAS* restrictive allograft syndrome

### Subgroup analysis for the occurrence of BOS and RAS

On CTi, no significant association between the criteria was analyzed and the occurrence of BOS was found. On CTf, a mosaic attenuation pattern was a risk factor for BOS (HR = 4.1 [CI: 1.4–12.5], *p* = 0.01), with a sensitivity of 40% and a specificity of 79% for this sign to predict the occurrence of BOS. Consolidations (with a threshold ≥ 4 segments) and pleural effusions were risk factors for BOS occurrence (HR = 2.6 [CI: 1.3–5.4], *p* = 0.01 and HR = 5.7 [CI: 1.4–22.3], *p* = 0.01, respectively). The univariate analyses did not reveal any potential predictive factor for RAS in this population. Due to the small number of RAS event, the model used in multivariate analyses was non-convergent.

### Subgroup analysis for the occurrence of death

On CTf, the presence of condensations (1–3 segments) and pleural effusions (HR = 8.2 [CI: 1.5–43.3], *p* = 0.029 and HR = 9.8 [CI: 2.7–35.7], *p* = 0.001, respectively) were significant risk factors for death (Table 3).

## Discussion

This retrospective, monocentric study shows the predictive role of early chest CT for the follow-up of LTx patients. In the first 4 months, moderate pulmonary artery stenosis was a risk factor for CLAD (HR = 4.5; CI [1.6–13.2]), and during the follow-up of at least 9 months after LTx, pleural effusion and consolidation were risk factors for CLAD and death.

Currently, the median survival after lung transplantation is 6.7 years overall and 8.9 years for patients who survive the first year. These results have not improved significantly over the last decade, in comparison with the previous decade, and the leading cause of late death remains CLAD (> 40% of cases) [26]. The diagnosis of CLAD is based on spirometry data and could be made once respiratory functional deterioration has set in. There is no treatment to recover normal lung function. Given the lack of effective treatment options for patients with known CLAD, prevention could be an attractive approach. The search for CT imaging risk factors predictive of CLAD occurrence during follow-up has not been very helpful in previous studies: CT changes could not predict survival in RAS patients; the sensitivities of air trapping, mosaic perfusion, bronchiectasis and bronchial wall thickening was low between 4 and 64%; and a composite CT score of the previous mentioned signs predict with low accuracy the worsening of FEV_1_ [3, 15, 16].

The presence of moderate pulmonary artery stenosis was significantly associated with the occurrence of CLAD. To the best of our knowledge, the link between pulmonary artery anastomosis, stenosis, and the occurrence of CLAD has not been evaluated in the literature thus far. The hypothesis of lung graft hypoperfusion in the pathophysiological mechanism of CLAD is an interesting field to explore both in link with pulmonary arterial stenosis or the non-restoration of bronchial arteries. This is the first study to show a link between pulmonary artery stenosis and CLAD, while other imaging sign on CT has already been described as aid diagnosis of CLAD [27]. The new era of dynamic contrast-enhanced MRI or perfusion CT with large row detectors could bring new elements on lung graft perfusion.

A mosaic pattern was present on the follow-up CT scan in 40% of BOS patients. Sensitivity remained low, probably due to the “early” nature of the first CT and the relatively low number of CLAD. Interestingly, the absence of mosaic on follow-up CT scans was predictive of good prognosis. This is in agreement with several studies that have shown a similarly low sensitivity (36 to 71%) and high specificity (78 to 100%) of mosaic for the occurrence of BOS [11, 12, 15]. Konen et al. included 52 transplanted patients (26 BOS and 26 non-BOS) and assessed the presence of mosaic, air trapping, bronchial thickening, and bronchial dilatation before and after the diagnosis of BOS. They showed that expiratory air trapping was the best predictor of BOS; however, in this study mosaic, bronchial wall thickening and bronchiectasis had a low sensitivity. Mosaic had a specificity close to 100% prior to spirometry diagnosis, which was confirmed by our results on a larger cohort [15].

Air trapping has been suggested in several publications as the most sensitive and specific CT sign for the early detection of BOS [13, 14, 17]. However, CT was performed late in the course of the disease, i.e., almost synchronously with the first signs of BOS, which creates a stark contrast with our study, as CT scans were performed early—on average 502 days before the diagnosis of BOS. The study by Lee et al. estimated that air trapping had a sensitivity of 74% and a specificity of 67% for the diagnosis of BOS; again, CT scanning was performed at the time of BOS diagnosis [17]. In this study, air trapping was not a risk factor for BOS occurrence. We can hypothesize that air trapping does not occur early (before 1 year) in the evolution of BOS patients because of a non-significant bronchial obstruction. In most cases, 22/29 patients with mosaic pattern on inspiratory CT have no air trapping on expiratory. In 7 cases, patients have both mosaic pattern on inspiratory CT and air trapping on expiratory CT, but the percentage of lung air trapping was in every case lower than the percentage of lung mosaic pattern. These abnormalities reinforce the role of the pulmonary artery stenosis.

There was a significant association between lung consolidation on CT and the occurrence of BOS, as well as pleural effusion. Usually, these imaging features are associated with lung infection, in association with ground-glass opacities, bronchial micronodules, and fuzzy micronodules. The latter were not associated with the occurrence of BOS. Consolidations are often considered trivial after LTx. Several studies have shown that acute rejection after LTx is a risk factor for BOS [28, 29]. Acute rejection can present with consolidations and pleural effusions [13]. This could explain the predictive role of consolidations and pleural effusion for CLAD in this study. The evolution of LTx is often marked by recurrent fungal or bacterial infections, which appear to be risk factors for the occurrence of BOS [30] and are often sided by consolidations. A recent study showed that severe primary graft dysfunction was associated with atelectasis on CT 3 months after transplantation but not with CLAD [31]; similar to the results presented, retractile consolidation on CTi was not a risk factor for CLAD.

Patient age and the indication for LTx were not risk factors for CLAD, confirming the findings of previous studies [20]. The presence of a bronchial anastomotic complication, although few such cases occurred, was not associated with the occurrence of CLAD and therefore does not jeopardize the long-term future of the graft.

There are some limitations to this study. There is a lack of power due to the design of a retrospective, single-center study, and the low number of RAS patients. Then, there are some methodological biases: intra- and interobserver variability was not assessed; several studies have shown good intra- and interobserver agreement for air trapping [16]; we did not correlate CT scores with graft dysfunction before CLAD, meaning patients with deterioration of FEV_1_ less than 20%; the CLAD definition is less reliable using lung function tests in patients with single lung transplantation (22.9%) and could be attributed not only to the decline in the transplanted single lung, but also to the decay in the not transplanted lung; primary graft dysfunction was not included in the statistical model despite being a risk factor for CLAD. Finally, about the score as the CTi was performed with a low degree of inspiration to obtain a good enhancement of the anastomoses and avoid flow artifacts due to Valsalva maneuver, this could have decreased the quality of the parenchymal analysis.

In conclusion, these results show a potential predictive role of chest CT in the follow-up of LTx patients for CLAD. Early chest CT should focus on pulmonary artery stenosis as this was a risk factor for CLAD in this study. During the follow-up (at least 9 months post-LTx), parenchymal consolidations and pleural effusions were shown to be risk factors for CLAD, and death in subgroup analyses.

### Supplementary Information

**Additional file 1.** 

## Data Availability

Data are available on reasonable request.

## Abbreviations

BOS: Bronchiolitis obliterans syndrome
CLAD: Chronic lung allograft dysfunction
CT: Computed tomography
FEV_1_: Percent predicted forced expiratory volume in 1 second
FVC: Forced vital capacity
LTx: Lung transplantation
RAS: Restrictive allograft syndrome
TLC: Total lung capacity

## References

[1] Chambers DC, Perch M, Zuckermann A, Cherikh WS, Harhay MO, Hayes D (2021). The International Thoracic Organ Transplant Registry of the International Society for Heart and Lung Transplantation: Thirty-eighth adult lung transplantation report — 2021; Focus on recipient characteristics. J Heart Lung Transplant.

[2] Sato M, Waddell TK, Wagnetz U, Roberts HC, Hwang DM, Haroon A (2011). Restrictive allograft syndrome (RAS): a novel form of chronic lung allograft dysfunction. J Heart Lung Transplant.

[3] Verleden SE, de Jong PA, Ruttens D, Vandermeulen E, van Raemdonck DE, Verschakelen J (2014). Functional and computed tomographic evolution and survival of restrictive allograft syndrome after lung transplantation. J Heart Lung Transplant.

[4] Verleden SE, Von Der Thüsen J, Van Herck A et al (2020) Identification and characterization of chronic lung allograft dysfunction patients with mixed phenotype: a single-center study. Clin Transplant 34(2) 10.1111/ctr.13781 31958356

[5] Yousem SA, Berry GJ, Cagle PT, Chamberlain D, Husain AN, Hruban RH (1996). Revision of the 1990 working formulation for the classification of pulmonary allograft rejection: Lung Rejection Study Group. J Heart Lung Transplant.

[6] Chamberlain D, Maurer J, Chaparro C, Idolor L (1994). Evaluation of transbronchial lung biopsy specimens in the diagnosis of bronchiolitis obliterans after lung transplantation. J Heart Lung Transplant.

[7] Kramer MR, Stoehr C, Whang JL, Berry GJ, Sibley R, Marshall SE (1993). The diagnosis of obliterative bronchiolitis after heart-lung and lung transplantation: low yield of transbronchial lung biopsy. J Heart Lung Transplant.

[8] Verleden GM, Raghu G, Meyer KC, Glanville AR, Corris P (2014). A new classification system for chronic lung allograft dysfunction. J Heart Lung Transplant.

[9] Taylor DO, Edwards LB, Boucek MM, Trulock EP, Keck BM, Hertz MI (2004). The Registry of the International Society for Heart and Lung Transplantation: twenty-first official adult heart transplant report–2004. J Heart Lung Transplant.

[10] Estenne M, Hertz MI (2002). Bronchiolitis obliterans after human lung transplantation. Am J Respir Crit Care Med.

[11] Leung AN, Fisher K, Valentine V, Girgis RE, Berry GJ, Robbins RC (1998). Bronchiolitis obliterans after lung transplantation: detection using expiratory HRCT. Chest.

[12] Siegel MJ, Bhalla S, Gutierrez FR, Hildebolt C, Sweet S (2001). Post-lung transplantation bronchiolitis obliterans syndrome: usefulness of expiratory thin-section CT for diagnosis. Radiology.

[13] Worthy SA, Park CS, Kim JS, Müller NL (1997). Bronchiolitis obliterans after lung transplantation: high-resolution CT findings in 15 patients. AJR Am J Roentgenol.

[14] Bankier AA, Van Muylem A, Knoop C, Estenne M, Gevenois PA (2001). Bronchiolitis obliterans syndrome in heart-lung transplant recipients: diagnosis with expiratory CT. Radiology.

[15] Konen E, Gutierrez C, Chaparro C, Murray CP, Chung T, Crossin J (2004). Bronchiolitis obliterans syndrome in lung transplant recipients: can thin-section CT findings predict disease before its clinical appearance?. Radiology.

[16] de Jong PA, Dodd JD, Coxson HO, Storness-Bliss C, Paré PD, Mayo JR (2006). Bronchiolitis obliterans following lung transplantation: early detection using computed tomographic scanning. Thorax.

[17] Lee ES, Gotway MB, Reddy GP, Golden JA, Keith FM, Webb WR (2000). Early bronchiolitis obliterans following lung transplantation: accuracy of expiratory thin-section CT for diagnosis. Radiology.

[18] Loubeyre P, Revel D, Delignette A, Wiesendanger T, Philit F, Bertocchi M (1995). Bronchiectasis detected with thin-section CT as a predictor of chronic lung allograft rejection. Radiology.

[19] Hemmert C, Ohana M, Jeung MY, Labani A, Dhar A, Kessler R (2014). Imaging of lung transplant complications. Diagn Interv Imaging.

[20] Saito T, Horie M, Sato M, Nakajima D, Shoushtarizadeh H, Binnie M (2016). Low-dose computed tomography volumetry for subtyping chronic lung allograft dysfunction. J Heart Lung Transplant.

[21] Verleden SE, Ruttens D, Vandermeulen E, Bellon H, Van Raemdonck DE, Dupont LJ (2015). Restrictive chronic lung allograft dysfunction: Where are we now?. J Heart Lung Transplant.

[22] Verleden GM, Glanville AR, Lease ED, Fisher AJ, Calabrese F, Corris PA (2019). Chronic lung allograft dysfunction: definition, diagnostic criteria, and approaches to treatment-A consensus report from the Pulmonary Council of the ISHLT. J Heart Lung Transplant.

[23] Levy L, Huszti E, Renaud-Picard B, Berra G, Kawashima M, Takahagi A (2020). Risk assessment of chronic lung allograft dysfunction phenotypes: validation and proposed refinement of the 2019 International Society for Heart and Lung Transplantation classification system. J Heart Lung Transplant.

[24] Hansell DM, Bankier AA, MacMahon H, McLoud TC, Müller NL, Remy J (2008). Fleischner society: glossary of terms for thoracic imaging. Radiology.

[25] Krishnam MS, Suh RD, Tomasian A, Goldin JG, Lai C, Brown K (2007). Postoperative complications of lung transplantation: radiologic findings along a time continuum. Radiographics.

[26] Chambers DC, Cherikh WS, Harhay MO, Hayes D, Hsich E, Khush KK (2019). The International Thoracic Organ Transplant Registry of the International Society for Heart and Lung Transplantation: Thirty-sixth adult lung and heart–lung transplantation Report—2019; Focus theme: Donor and recipient size match. J Heart Lung Transplant.

[27] Kim SJ, Azour L, Hutchinson BD, Shirsat H, Zhou F, Narula N (2021). Imaging course of lung transplantation: from patient selection to postoperative complications. RadioGraphics.

[28] Verleden SE, Ruttens D, Vandermeulen E, Vaneylen A, Dupont LJ, Van Raemdonck DE (2013). Bronchiolitis obliterans syndrome and restrictive allograft syndrome: do risk factors differ?. Transplantation.

[29] Davis WA, Finlen Copeland CA, Todd JL, Snyder LD, Martissa JA, Palmer SM (2012). Spirometrically significant acute rejection increases the risk for BOS and death after lung transplantation. Am J Transplant.

[30] Glanville AR, Gencay M, Tamm M, Chhajed P, Plit M, Hopkins P (2005). Chlamydia pneumoniae infection after lung transplantation. J Heart Lung Transplant.

[31] Li D, Abele J, Weinkauf J et al (2021) Atelectasis in primary graft dysfunction survivors after lung transplantation. Clin Transplant 35(7) 10.1111/ctr.14315 33848359

