# Supplementary material for: Early chest CT abnormalities to predict the subsequent occurrence of chronic lung allograft dysfunction
Source: Insights Imaging. 2023 Sep 23;14:154. doi: 10.1186/s13244-023-01509-3 (PMC10517910; doi:10.1186/s13244-023-01509-3)
Supplement: Supplementary file 1 — Additional file 1. [file 13244_2023_1509_MOESM1_ESM.docx]

**Early chest CT abnormalities to predict the subsequent occurrence of chronic lung allograft dysfunction**

**ELECTRONIC SUPPLEMENTARY MATERIAL**

**Table e1:**

|  | **stables* N=93** | **CLAD N=25** | | **BOS N=19** | | **RAS N=6** | | **death N= 19** | |
| --- | --- | --- | --- | --- | --- | --- | --- | --- | --- |
|  |  |  | *p* |  | *p* |  | *p* |  | *p* |
| **Age at LTx (years)** | 44 ± 15 | 46 ± 15 | 0.63 | 49 ± 14 | 0.18 | 39 ± 16 | 0.36 | 46 ± 14 | 0.57 |
| **Gender (male)** | 45 (48%) | 18 (69%) | 0.06 | 15 (75%) | 0.03 | 3 (50%) | 0.71 |  |  |
| **Recipient TLC before LTx (%)** | 99 ± 43 | 105 ± 36 | 0.53 | 105 ± 37 | 0.59 | 98 ± 37 | 0.91 | 102 ± 35 | 0.75 |
| **Theoretical donor TLC (L)** | 6.0 ± 1.1 | 6.4 ± 1.3 | 0.11 | 6.6 ± 1.3 | 0.03 | 5.4 ± 1.5 | 0.08 | 6.1 ± 1.1 | 0.85 |
| **FEV_1_ baseline (L)** | 2.6 ± 0.9 | 2.7± 0.9 | 0.68 | 2.6 ± 0.8 | 0.87 | 3.2 ± 1.8 | 0.36 | 2.6 ± 0.9 | 0.95 |
| **Aetiology for LTx** |  |  | 0.16 |  | 0.10 |  | 0.45 |  | 0.26 |
| Emphysema | 25 (27%) | 10 (40%) | | 9 (47%) | | 1 (17%) | | 7 (37%) | |
| Fibrosis | 22 (24%) | 6 (24%) | | 4 (21%) | | 2 (33%) | | 3 (16%) | |
| CF and bronchial dilatations | 37 (40%) | 5 (20%) | | 3 (16%) | | 2 (33%) | | 5 (26%) | |
| New LTx for CLAD | 5 (5%) | 1 (4%) | | 1 (5%) | | 0 (0%) | | 1 (5%) | |
| Others | 4 (4%) | 3 (12%) | | 2 (11%) | | 1 (17%) | | 3 (16%) | |

Note: clinical description of the stable, CLAD, BOS, RAS, or deceased populations. Results are given as mean ± standard deviation or n (%). *Stable: correspond to non-CLAD patients. P-value compared each group with the stable group. BOS: bronchial obstruction syndrome; CLAD: chronic lung allograft disease; LTx: lung transplantation; RAS: restrictive allograft disease.

**Table e2:**

| **Tree in bud nodules** *(score on 20)* | 1.2 [0-10] |
| --- | --- |
| **Ground-glass nodules** *(score on 20)* | 1.4 [0-12] |
| **Bronchial wall thickening** *(score on 20)* | 2.4 [0-20] |
| **Bronchial wall thickening** (*0/1/2)* | 76 (64%) /40 (34%)/ 2 (2%) |
| **Mucous plugs** *(score on 20)* | 1.39 [0-16] |
| **Proximal bronchiectasis** *(score on 20)* | 0.46 [0-10] |
| **Bronchiectasis severity** *(score 0/1/2/4)* | 108(89%)/2(2%)/5(4.1%)/3(3%) |
| **Distal bronchiectasis** *(score on 20)* | 1.3 [0-9] |
| **Mosaic attenuation** *(yes/no)* | 13(11%)/105(89%) |
| **Ground-glass opacities** (*0/1/2/3)* | 78(66%)/28(24%)/8(7%)/1(1%)/3(2%) |
| **Consolidations** (*0/1/2/3/4)* | 23(20%)/37(31%)/39(33%)/15(13%)/4(3%) |
| **Interstitial opacities** *(0/1/2/3/4)* | 27(23%)/38(32%)/29(25%)/12(10%)/12(10%) |
| **Pleural effusion** (*0/1/2/3)* | 63(53%)/44(37%)/11(9%)/0(0%) |
| **Pneumothorax** (*0/1/2/3)* | 98(83%)/16(14%)/3(3%)/1(1%) |
| **Anastomotic granuloma** *(yes/no)* | 93(78%)/25(20%) |
| **Bronchial anastomosis stenosis** |  |
| - right = *0/1/2* | 101(86%)/2(2%)/0(0%) |
| - left *0/1/2* | 96(81%)/9(7%)/3(2%) |
| **Bronchial anastomotic dehiscence** |  |
| - right *(0/1/2)* | 86(73%)/10(9%)/7(6%) |
| - left *(0/1/2)* | 100(85%)/3(3%)/2(2%) |
| **Total lung volume (L)** | 3.2 [1.2- 6.0] |
| **Pulmonary embolism** *0/1/2/3* | 99(84%)/9(8%)/10(8%)/0(0%) |
| **Arterial anastomotic stenosis** |  |
| - right *(0/1/2)* | 87(74)/5(4%)/0(0%) |
| - left *(0/1/2)* | 83(70%)/11(9%)/3(3%) |

Note: Initial CT data (CTi) on the total population. Results as mean [min-max] or n (%).

**Table e3:**

| **Tree in bud nodules** *(score on 20)* | 0.42 [0-13] |
| --- | --- |
| **Ground-glass nodules** *(score on 20)* | 0.58 [0-12] |
| **Bronchial wall thickening** *(score on 20)* | 2.19 [0-20] |
| **Bronchial wall thickening** (*0/1/2)* | 89(75%)/26(22%)/3(2%) |
| **Mucous plugs** *(score on 20)* | 0.69 [0-12] |
| **Proximal bronchiectasis** *(score on 20)* | 0.68 [0-11] |
| **Distal bronchiectasis** *(score on 20)* | 0.75 [0-10] |
| **Mosaic attenuation** *(yes/no)* | 29(25%)/89(75%) |
| **Ground-glass opacities** (*0/1/2/3)* | 91(77%)/16(14%)/6(5%)/3(3%)/2(2%) |
| **Consolidations** (*0/1/2/3/4)* | 61(52%)/50(42%)/5(4%)/2(2%)/0(0%) |
| **Interstitial opacities** *(0/1/2/3/4)* | 28(24%)/55(47%)/25(21%)/4(5%)/6(3%) |
| **Pleural effusion** (*0/1/2/3)* | 108(92%)/10(9%)/0(0%)/0(0%) |
| **Anastomotic granuloma** *(yes/no)* | 85(72%)/31(26%) |
| **Bronchial anastomosis stenosis** |  |
| - right = *0/1/2* | 99(84%)/2(2%)/0(0%) |
| - left *0/1/2* | 100(85%)/4(3%)/1(1%) |
| **Bronchial anastomotic dehiscence** |  |
| - right *(0/1/2)* | 88(75%)/10(9%)/4(3%) |
| - left *(0/1/2)* | 96(81%)/9(8%)/0(0%) |
| **Total lung volume (L)** | 4.3 [1.6-7.1] |
| **Delta volume (L)** | 1.1 [0.4-1.1] |

Note: Follow-up CT data (CTf) on the total population. Results as mean [min-max] or n (%).
